# A Scoping Review of Creatine Supplementation in Alzheimer’s Disease: Evidence from Preclinical and Clinical Studies

**DOI:** 10.3390/nu18213477

**Published:** 2026-10-23

**Authors:** Javier Choquet de Isla, Javier Riscart-López, Ismael Sánchez-Gomar, Manuel Bández-Ruiz, Ignacio Rosety-Rodríguez, Francisco Brenes-Martín, Nicolas G. Bazán, Elkin Navarro Quiroz, Miguel Ángel Rosety-Rodríguez, Noelia Geribaldi-Doldán

**Affiliations:** 1 Department of Didactics of Physical Education, Art and Music, Area of Physical and Sports Education, Faculty of Education, University of Cadiz, 11519 Puerto Real, Spain; javier.choquet@uca.es; 2 Biomedical Research and Innovation Institute of Cádiz (INIBICA), 11002 Cádiz, Spain; javier.riscart@uca.es (J.R.-L.); ismael.sanchez@uca.es (I.S.-G.); manuel.bandez@uca.es (M.B.-R.); ignacio.rosety@uca.es (I.R.-R.); noelia.geribaldi@gm.uca.es (N.G.-D.); 3 Department of Medicine, Faculty of Medicine, University of Cadiz, 11002 Cádiz, Spain; 4 Department of Biomedicine, Biotechnology and Public Health, Area of Physiology, Faculty of Medicine, University of Cadiz, 11002 Cádiz, Spain; 5 Department of Biomedicine, Biotechnology and Public Health, Area of Biochemistry, Faculty of Medicine, University of Cadiz, 11002 Cádiz, Spain; 6 Department of Basic Medical Science, Area of Human Anatomy and Embryology, Faculty of Medicine, University of Cadiz, 11002 Cádiz, Spain; 7 Neuroscience Center of Excellence, School of Medicine, Louisiana State University Health New Orleans, New Orleans, LA 70112, USA; 8 Faculty of Basic and Biomedical Sciences, Center for Research in Life Sciences, Universidad Simón Bolívar, Barranquilla 080002, Colombia

**Keywords:** ageing, Alzheimer’s disease, neurodegeneration, creatine, nutrition, bioenergetics, preclinical models

## Abstract

**Background/Objectives:** Although creatine supplementation has a theoretical rationale for neuroprotection, public interest has outpaced available scientific evidence. Alzheimer’s disease (AD), a major and growing global health concern, is among the neurodegenerative diseases proposed to benefit from creatine supplementation. This scoping review systematically maps the existing literature on creatine supplementation for AD to identify gaps that limit translational progress. **Methods:** Following JBI methodology and PRISMA-ScR guidelines, a search in five electronic databases was conducted for preclinical and clinical studies evaluating creatine interventions in AD. The methodological quality of the studies was appraised using the SYRCLE and NIH tools, respectively. **Results:** Nine unique studies were included. The preclinical models investigated neuron apoptosis, oxidative stress, neuroinflammation, mitochondrial function, cognitive outcomes, and pathophysiological clearance. Conversely, clinical studies were limited to a pilot trial that suggested preliminary tolerability but could not establish causality. This review identifies six limitations within the current evidence: (1) a reliance on acute or non-transgenic animal models that lack construct validity; (2) a need to address sexual dimorphism; (3) a lack of studies exploring administration routes to bypass the blood–brain barrier; (4) a complete lack of independent randomized controlled trials (RCTs), with all clinical evidence originating from a single research group; (5) an absence of research assessing creatine as a preventive strategy; and (6) a shortage of assessment regarding physical syndromes, such as frailty. **Conclusions:** The scientific evidence for creatine in AD remains preliminary. Future research should prioritize progressive transgenic models, evaluate noninvasive administration routes, investigate effects on frailty, assess long-term preventive efficacy, account for sex as a variable, and transition to rigorous RCTs.

## 1. Introduction

Creatine (α-methylguanidinoacetic acid) is a naturally occurring compound synthesized endogenously from reactions involving the amino acids arginine, glycine and methionine. These reactions primarily take place in the kidneys, liver and pancreas. However, the brain also possesses the capacity for de novo creatine synthesis. Exogenously, creatine is primarily consumed through meat or as a dietary supplement. Its primary mechanism of action involves the rapid provision of energy by resynthesizing adenosine triphosphate (ATP) [1,2,3,4].

Building on this physiological foundation, creatine monohydrate (CrM) is one of the most widely consumed dietary supplements globally and is supported by one of the most extensive bodies of scientific evidence, both in athletic performance and general health contexts. It remains one of the few supplements to consistently demonstrate robust ergogenic and therapeutic benefits alongside a highly favorable safety profile, across diverse genders, ages, and health conditions [2,3,5,6,7,8,9,10,11,12]. In fact, it has been proposed as a ‘conditionally essential nutrient’ given that the quantity of creatine the body naturally synthesizes is often insufficient to meet human needs. Consequently, humans must obtain adequate creatine from diet to fulfill this requirement [13]. Indeed, the International Society of Sports Nutrition has adopted the position that government legislatures and sport organizations who restrict or discourage use of creatine may be placing athletes at greater risk, particularly in contact sports that carry a risk of head trauma or neurological injury, thereby opening themselves up to legal liability [5].

While its ergogenic effects on sport performance and body composition are widely documented across different populations [3,11,14,15,16,17,18,19,20,21,22], creatine supplementation is now generating considerable scientific interest for its benefits outside of athletic contexts, primarily in the domain of cognitive function and memory [1,23,24,25,26].

Similar to skeletal muscle, the brain is an organ with an extremely high energy demand that relies critically on the phosphocreatine (PCr) system to maintain energy homeostasis and rapid ATP recycling. Research has shown that dietary creatine is actively transported across the blood–brain barrier (BBB) by the creatine transporter, increasing cerebral PCr stores and thereby improving neuronal metabolic capacity [24,25,26,27,28]. Parallel to these cerebral bioenergetic enhancements, creatine supplementation has been reported to modulate lipid profiles (lowering cholesterol and triglycerides), exhibit antioxidant activity, exert anti-inflammatory and immunomodulatory effects, and enhance glycemic control, among other general health benefits [29,30,31]. Together, these central and systemic mechanisms likely underpin why creatine may confer beneficial cognitive effects, with the strongest evidence observed in the domains of memory, reasoning, attention, and information processing speed [23,32,33,34].

These neurocognitive effects appear most pronounced under conditions of metabolic stress (such as sleep deprivation or hypoxia) or in populations with reduced baseline creatine levels (like vegetarians or older adults). This underscores the importance of research that accounts for pre- and post-supplementation brain creatine content, as well as novel administration routes designed to enhance brain creatine levels [25,35,36,37,38,39,40,41].

In this context, creatine supplementation has been suggested as a potentially beneficial approach for various neurodegenerative and neuropsychiatric conditions, including Parkinson’s disease, Huntington’s disease, Alzheimer’s disease (AD), amyotrophic lateral sclerosis, and traumatic brain injury, as well as depression and anxiety [42,43,44,45,46,47,48]. However, the strong theoretical basis has not been consistently translated into robust clinical findings. Indeed, the available evidence is limited, and existing systematic reviews and meta-analyses of some of these conditions have demonstrated only small effects, which are often lacking in clear clinical relevance [49,50,51,52].

Among neurodegenerative diseases, AD is a major and growing global health concern as it is the leading cause of dementia [53]. This condition affected around 57 million people worldwide in 2021, with almost 10 million new cases occurring each year [54]. Driven by a rapidly aging global population, projected to increase from 1 billion individuals aged 60 and over in 2019 to 2.1 billion by 2050, this epidemiological burden is expected to surge substantially [55]. In the United States, an estimated 7.2 million individuals aged 65 and older are living with AD as of 2025, a figure projected to reach 13.8 million by 2060. Consequently, associated annual healthcare and long-term care costs are expected to escalate from $384 billion in 2025 to nearly $1 trillion by 2050 [56]. Europe faces a comparable challenge: while a 2017 meta-analysis established an AD prevalence of 5.05% and an incidence of 11.08 per 1000 person-years [57], recent estimates for 2025 report over 12.1 million people living with dementia across the continent (including 9.1 million within the EU-27). By 2050, this European demographic is projected to approach 20 million [58].

Underpinning this epidemiological burden, AD is a progressive, complex, and multifactorial neurodegenerative disorder that accounts for most dementia cases globally [54,59,60]. Currently understood as a biological continuum, AD initiates as a silent process driven by early neuropathological changes while individuals are still asymptomatic [61]. This underlying pathogenesis is primarily characterized by the increased production and deposition of amyloid-beta (Aβ) plaques and the abnormal phosphorylation of tau protein into neurofibrillary tangles (NFTs). As this neuropathological burden progresses, it triggers a cascade of detrimental cellular events, including elevated levels of reactive oxygen species (ROS), mitochondrial dysfunction, loss of synaptic plasticity, and subsequent neuronal cell death in specific brain regions [54,59,60,61]. Ultimately, this profound biological damage manifests as a fatal, age-associated trajectory of cognitive and functional decline [60]. The clinical presentation encompasses a broad spectrum of debilitating symptoms, most notably memory loss that disrupts daily life, disorientation regarding time and place, and challenges in executive planning or problem-solving. As the disease advances, patients typically exhibit difficulties completing familiar tasks, visuospatial deficits, linguistic impairments, poor judgment, and profound changes in mood, personality, and behavior, frequently leading to complete withdrawal from occupational and social activities [56].

Currently approved therapies include cholinesterase inhibitors, NMDA-receptor antagonists and monoclonal antibodies. However, these medications were gradually discovered to yield modest clinical benefits and are constrained by significant adverse effects, having only symptomatic effects [56,59,62]. Concurrently, non-pharmacological interventions are widely utilized to manage AD symptoms. In some instances, these approaches demonstrate greater efficacy than conventional medications while posing minimal risk or harm to patients [56]. Given these clinical limitations, ongoing research is pursuing novel therapeutic targets and alternative pathophysiological frameworks, placing interventions like creatine supplementation firmly in the spotlight.

The scientific rationale for investigating creatine specifically stems from an ongoing paradigm shift in understanding the disease. While classically characterized by the presence of Aβ plaques and NFTs, AD is increasingly recognized as a metabolic disorder driven by early and progressive cerebral glucose hypometabolism and mitochondrial dysfunction. This underlying ‘bioenergetic crisis’ leads to chronic ATP depletion, rendering neurons highly vulnerable to oxidative stress and excitotoxicity [63,64,65,66,67,68,69,70,71,72,73]. Crucially, this metabolic impairment is accompanied by significantly reduced levels of creatine and PCr in the brains of AD patients compared to healthy age-matched controls, a finding that also suggests its potential utility as a biomarker [69,74,75,76]. Furthermore, a recent multi-omics investigation utilizing two-sample Mendelian randomization has robustly substantiated this relationship, identifying elevated cerebrospinal fluid creatine levels as a causal protective factor against AD risk [77]. Within this context, creatine supplementation is hypothesized to exert neuroprotective effects not only by buffering cellular energy reserves but also by enhancing mitochondrial function [78,79,80,81,82], mitigating oxidative stress [83,84,85], and potentially attenuating Aβ and glutamate-induced toxicity [81,86].

Although the therapeutic potential of creatine in neurodegenerative diseases is a subject of active research, public and commercial interest has surged. This interest, manifested in rising sales and social media engagement, appears to have prematurely outpaced the limited supporting scientific literature. This discrepancy risks misleading consumers and undermining informed decision-making [87]. This highlights the urgent need to establish the current state of evidence.

Therefore, the aim of this scoping review is to systematically map the extent, range, and nature of research on creatine supplementation for AD, encompassing clinical trials and relevant preclinical studies. Furthermore, this review is designed to synthesize the findings of the current evidence, pinpointing the gaps that currently restrict the translational potential of creatine as a strategy for AD.

## 2. Materials and Methods

### 2.1. Protocol and Registration

This scoping review was conducted in accordance with the methodological guidance of the Joanna Briggs Institute (JBI) [88], drawing on the framework first described by Arksey and O’Malley [89] and further refined by Peters et al. [90]. Reporting follows the Preferred Reporting Items for Systematic Reviews and Meta-Analyses extension for Scoping Reviews (PRISMA-ScR) checklist [91] (see Supplementary S1).

The protocol was registered on March 2026 with the Open Science Framework (OSF; 10.17605/OSF.IO/GCN4S).

### 2.2. Eligibility and Inclusion Criteria

Peer-reviewed articles published in English, Spanish, Portuguese or Italian, involving human participants or relevant animal models of AD, and directly assessing the effects of creatine supplementation were considered. Quantitative, qualitative, and mixed-method studies were included to capture different aspects of creatine’s supplementation impact.

Eligibility criteria were defined according to the Population, Concept, and Context (PCC) framework [88], including Types of evidence sources (T), as follows:

- Population: Studies including humans diagnosed with AD at any stage, as well as experimental animal models, were eligible. No restrictions were applied regarding age, sex, or disease severity.
- Concept: The central concept of interest was supplementation with creatine or its analogues, either alone or in combination with other interventions, as long as the specific effects of creatine could be assessed. Outcomes of interest included cognitive function, quality of life, neurobiological mechanisms, disease progression, safety, and tolerability. Key characteristics of the intervention, such as route of administration, dosage, and duration, will also be mapped.
- Context: Studies were eligible regardless of geographical region, cultural setting, or healthcare context. Both clinical settings and preclinical laboratory studies were included.
- Types of evidence sources: Published and unpublished primary research studies and experimental animal studies were considered. Grey literature, as well as conference abstracts and proceedings, were also included to ensure a broad range of evidence was captured. Review articles were excluded, as they do not present original data and do not contribute new primary evidence relevant to the research question.

### 2.3. Search Strategy

A comprehensive literature search was conducted in Embase (via Elsevier, Amsterdam, The Netherlands), PubMed (via NCBI, Bethesda, MD, USA), Web of Science (Core Collection, via Clarivate, Philadelphia, PA, USA), Scopus (via Elsevier, Amsterdam, The Netherlands), and PsycINFO (via ProQuest, Ann Arbor, MI, USA) from inception to 16 October 2025. A final updated search using the exact same strategy was performed on 14 August 2026, to identify any recently published articles prior to manuscript submission and the newly identified records were integrated into the final PRISMA flow diagram.

A systematic search was conducted combining terms related to AD (“Alzheimer *”, “Alzheimer’s disease”) with terms referring to creatine supplementation (“creatine”, “creatine monohydrate” and “creatine supplementation”). Both search topics were combined with the Boolean operators ‘AND’ (inter-topics) and ‘OR’ (intra-topics).

To ensure comprehensive coverage, additional manual searches were conducted using relevant keywords, and the reference lists of all included studies and pertinent reviews were screened for further eligible publications. This search was independently developed and executed by two investigators (J.C.d.I. and J.R.L.) under the oversight of a senior reviewer (N.G.D.). No temporal restrictions were applied.

The complete search strategies for all databases are available in Supplementary S2 and the published protocol to guarantee transparency and reproducibility.

### 2.4. Selection of Sources of Evidence and Data Charting

The study selection process was carried out in three stages by two researchers (J.C.d.I. and J.R.L.) independently. After each stage, the researchers compared their results and reached a consensus. Firstly, the results from all database searches were imported into the Rayyan QCRI software [92]. Rayyan automatically flagged potential duplicates, which were then carefully verified and removed manually by the reviewers. Next, both reviewers assessed the titles and abstracts of the selected articles based on the predefined eligibility and inclusion criteria. Finally, the full texts of the remaining articles were reviewed to determine their inclusion. In cases of disagreement at any stage, a third researcher (N.G.D.) was designated to make the final decision. The screening process and results of the literature search are summarized in a flowchart diagram, which was generated using the PRISMA2020 R package and ShinyApp tool by Haddaway et al. [93].

For data extraction, researchers (J.C.d.I. and J.R.L.) independently extracted the following information from each eligible original study using a standardized form, with items adapted to study type. For clinical studies, extracted information included (i) first author’s name and year of publication; (ii) country; (iii) study design; (iv) intervention duration, groups and their sample sizes; (v) administration route (vi) outcomes assessed; (vii) main results; and (viii) risk of bias. For preclinical studies, information extracted included (i) first author’s name and year of publication; (ii) animal species and disease model (including sex and age); (iii) experimental duration, groups and their sample sizes; (iv) administration route; (v) outcomes assessed; (vi) main results; and (vii) risk of bias. Discrepancies in data extraction were resolved through discussion among the researchers (including N.G.D.) until the consensus was reached.

### 2.5. Critical Appraisal of Individual Sources of Evidence

Although critical appraisal or risk of bias assessment is not mandatory in scoping reviews [88], an assessment of methodological quality was conducted in this review. This evaluation was not intended to exclude studies but to contextualize the extent to which specific gaps in the literature have been adequately addressed. In particular, assessing the quality of existing studies allows determining whether an identified gap has been meaningfully investigated or whether methodological limitations prevent drawing firm conclusions, leaving the gap unresolved.

Thus, methodological quality was assessed using SYRCLE’s risk-of-bias tool for animal studies [94] and the NIH Quality Assessment Tool for Before-After (Pre-Post) Studies with No Control Group for pilot trials in humans [95]. Detailed assessment tables are provided in Supplementary S3. To facilitate the interpretation of these assessments, risk-of-bias visualizations were generated using the robvis web-based tool [96]. Given the small number and heterogeneity of studies, the appraisal was used solely to contextualize whether reported findings sufficiently addressed the identified evidence gaps.

### 2.6. Synthesis of Results

Extracted data were organized and synthesized descriptively to map the current state of evidence on creatine supplementation in AD. Clinical and preclinical studies were analyzed separately, and findings were summarized in descriptive tables and narrative form, highlighting intervention characteristics, outcomes measured, and key results. Methodological quality and risk-of-bias assessments were considered to contextualize the robustness of the evidence and determine whether specific gaps in the literature have been addressed. The discussion will provide an in-depth evaluation of evidence gaps, illustrating outcomes that remain insufficiently studied and outlining methodological approaches necessary for future research to generate robust and clinically meaningful evidence on the effects of creatine in AD.

## 3. Results

**Figure 1:**
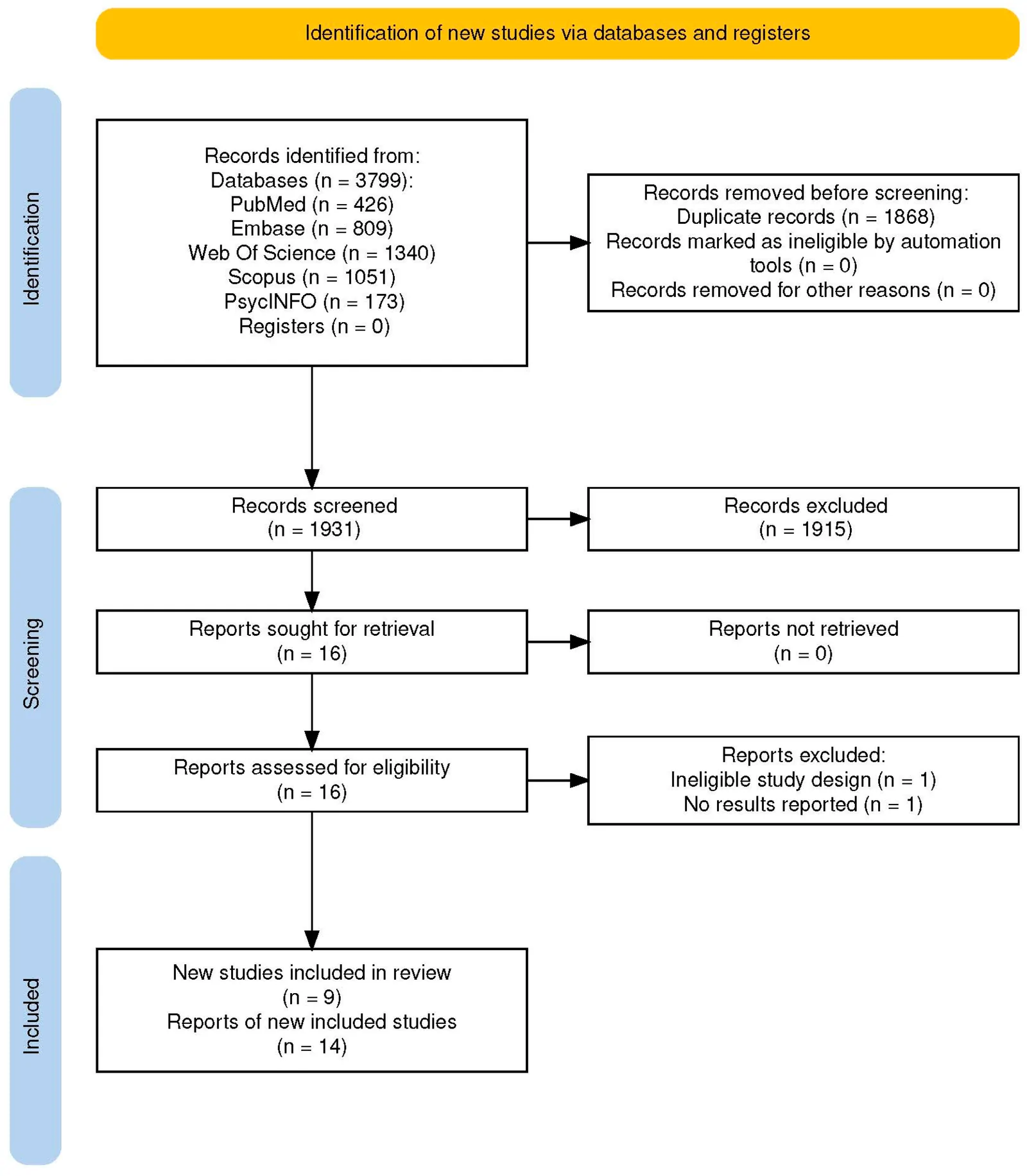
Flowchart depicting the study selection process. Diagram generated using the web-based tool by Haddaway et al. [93].

A total of 3799 articles were retrieved from the initial database searches. After duplicate removal, 1931 articles were screened at the title and abstract level. The overall inter-rater agreement for the title and abstract screening was 99.7% (kappa = 0.81). Disagreements at this stage were resolved by consensus, with mediation by the third reviewer. This process yielded 16 articles for full-text review. Of these, 2 were subsequently excluded due to an ineligible study design (in vitro model, *n* = 1) and a lack of reported outcomes (study protocol, *n* = 1). Consequently, 14 reports met the inclusion criteria. As one preclinical studies was represented by two reports, and the single clinical trial was represented by five reports, a total of 9 unique studies were included in this scoping review (see Figure 1).

Of the 9 unique studies meeting the inclusion criteria, 8 (88.9%) were conducted in experimental animal models, whereas 1 (11.1%) involved human participants. Overall, the synthesized literature exhibited substantial methodological heterogeneity. To facilitate a structured narrative synthesis, the extracted data were stratified into two distinct domains: preclinical evidence and clinical evidence. This bipartite approach allows for a clear delineation between the mechanistic pathways elucidated in controlled laboratory environments and the preliminary translational findings emerging from recent human pilot trials.

### 3.1. Preclinical Evidence in Animal Models of Alzheimer’s Disease

Among the animal studies, rodents were primarily utilized to explore oxidative stress (37.5%, *n* = 3) [97,98,99], various molecular and morphological mechanisms (75.0%, *n* = 6) [97,98,100,101,102,103], cognitive and behavioral effects (75.0%, *n* = 6) [97,99,100,101,102,103], mitochondrial function (12.5%, *n* = 1) [103], neuronal damage (50.0%, *n* = 4) [98,100,101,102], and muscle-related outcomes (12.5%, *n* = 1) [104] following creatine or PCr supplementation. The detailed characteristics, specific methodological approaches, and main findings of these preclinical investigations are summarized in Table 1.

The included preclinical studies spanned a decade of research, with the earliest reports appearing in 2015 [102,104]. Subsequent singular studies were published in 2016 [101], 2018 [99], 2020 [103], and 2021 [98]. Most recently, two additional studies were published in 2025, reflecting continued interest in this experimental domain [97,100].

Regarding the administration routes and dosing strategies, oral administration was the most prevalent method, with most studies providing creatine chronically via a supplemented diet at 2% or 3% (*w*/*w*) over a period ranging from 6 to 10 weeks (63%, *n* = 5) [97,101,102,103,104]. Interestingly, studies utilizing 3% supplemented diet consistently reported decreased cognitive deficits in the Morris water maze (MWM) [97,103], whereas those using a 2% supplemented diet reported either exacerbated or unchanged cognitive deficits [101,102]. Alternatively, one study employed acute oral administration via gavage to deliver a single dose of 0.01 or 10 mg/kg of creatine [99]. To bypass physiological barriers or administer specific formulations like PCr, other experimental designs utilized injectable routes. These included weekly intraventricular injections (0.125 mg) over 4 weeks [100] and weekly peripheral PCr injections (20 or 50 mg/kg) over the final 8 weeks of a 10-week protocol [98].

As regards intervention duration, the preclinical protocols exhibited significant variability in length, ranging from single-dose administrations to regimen lasting up to 10 weeks. Most of experimental designs used chronic supplementation models (87.5%, *n* = 7) [97,98,100,101,102,103,104]. Within these chronic protocols, the most prevalent duration was 8 weeks, implemented in 37.5% (*n* = 3) of the studies [97,103,104]. Ten-week interventions were used in 25% (*n* = 2) of the models [98,101], while 6-week [102] and 4-week [100] interventions accounted for 12.5% (*n* = 1) each. In contrast, only one study (12.5%, *n* = 1) used an acute, single-dose administration model to evaluate immediate neurobehavioral and biochemical responses [99]. When analyzing the administration timeline, five studies (62.5%) applied a therapeutic framework by administering creatine post-injury. Conversely, the remaining three studies (37.5%) implemented a preventive strategy, introducing supplementation either before or at the same time as the neurotoxic challenge.

Concerning cognitive and behavioral outcomes, creatine supplementation demonstrated primarily neuroprotective and cognitive-enhancing effects across various experimental models, although results varied. When assessing spatial learning and memory via the MWM, several studies reported significant improvements, such as decreased escape latency and increased time spent in the target quadrant, in both D-galactose-induced aging male mice [97] and female transgenic AD models [100]. Conversely, findings in male rat models of Aβ toxicity evaluated with the same MWM were mixed, reporting either no significant cognitive changes or exacerbated cognitive deficits following creatine supplementation [101,102]. Of particular interest regarding spatial cognition, Snow et al. [103] observed sex-specific responses in the 3xTg mouse model, where females exhibited improved performance while males showed altered spatial search strategies. Beyond memory, acute creatine administration also attenuated depressive-like behaviors and anhedonia in Aβ-injected mice [99].

Focusing on the underlying molecular and morphological mechanisms, creatine and PCr administration consistently mitigated hallmark AD pathologies across several models. Multiple studies reported significant reductions in Aβ burden or toxicity [100,103], alongside decreased expression of AD-associated molecular markers such as p-Tau, APP, and CDK5 [98]. Furthermore, creatine demonstrated anti-apoptotic and neuroprotective properties, significantly reducing neuronal damage in the hippocampus while simultaneously activating survival and astrocytic support pathways (e.g., AKT, GSK-3β, and GFAP) [98,100]. Interestingly, aligning with the behavioral findings, the 2% supplemented diet failed to elicit these robust molecular benefits, showing no significant changes in apoptotic neurons or Aβ plaques in Aβ-injected rats [101,102]. Finally, creatine exhibited antioxidant (e.g., SOD and glutathione (GSH)) [97,98] and anti-inflammatory effects (e.g., IBA1 and pSTAT1) [100], and promoted synaptic integrity; it significantly enhanced structural plasticity markers (e.g., PSD-95, BDNF) [97,103], reduced markers of oxidative stress (such as MDA) [97,98], and modulated the compensatory antioxidant response following neurotoxic insults [99]. Creatine supplementation also positively influenced mitochondrial and peripheral muscle bioenergetics. Specifically, Snow et al. [103] reported that creatine enhanced hippocampal mitochondrial function in female transgenic mice, evidenced by increased coupled respiration and coupling efficiency, indicating improved ATP production capacity. Furthermore, Farshidfar et al. [104] observed that dietary creatine successfully elevated creatine content and branched-chain amino acid levels, particularly leucine, in the quadriceps muscle of an AD mouse model, suggesting potential benefits for skeletal muscle function.

**Figure 2:**
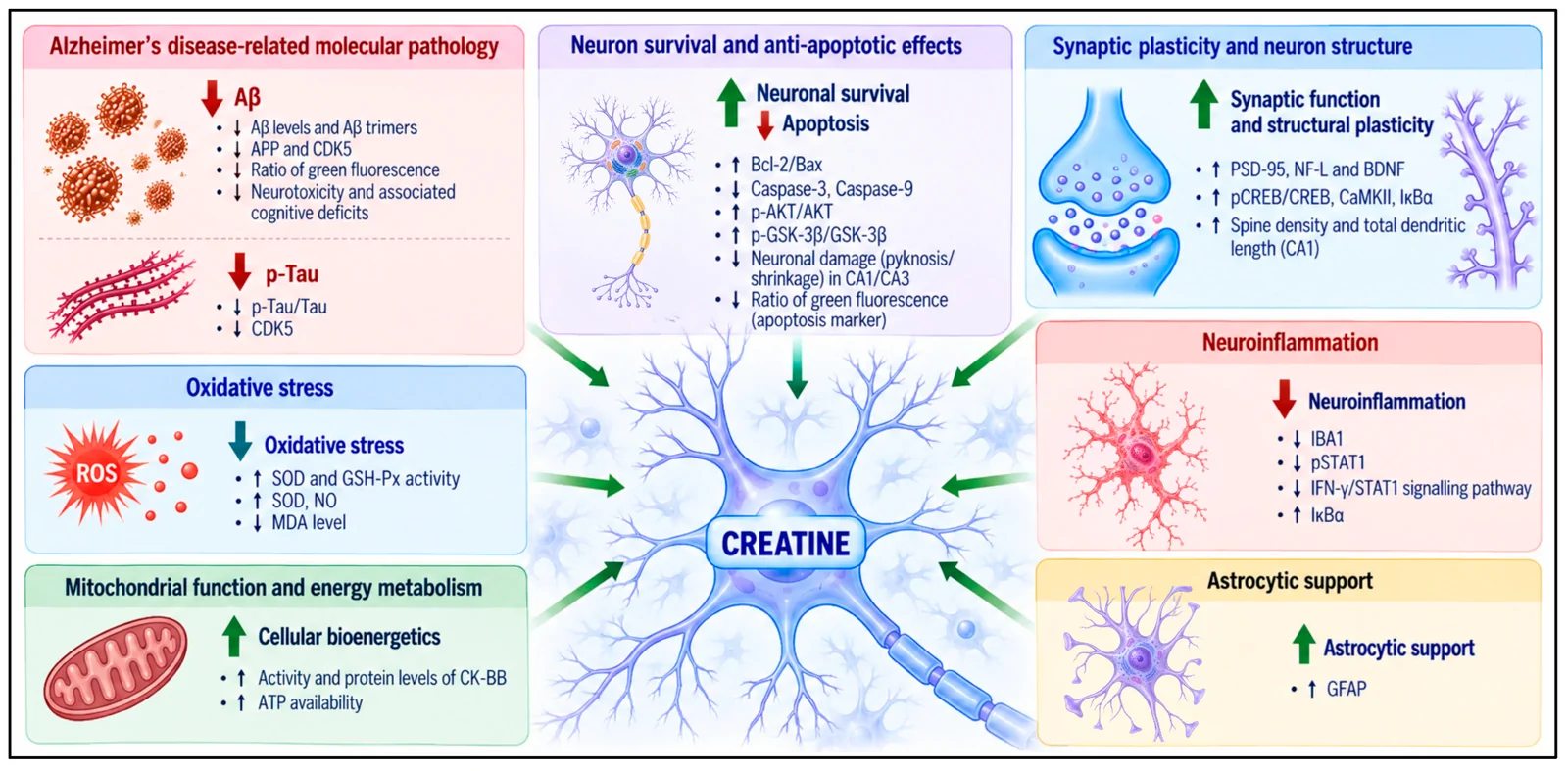
Graphical representation of the effects of creatine on nerve cells and Alzheimer’s disease-related molecular pathology. The arrows indicate: ↓ decreased; ↑ increased. Individual graphical elements were generated using ChatGPT (OpenAI, 2026) and subsequently arranged and composed by the authors using Canva (Canva Pty Ltd., Sydney, Australia).

To visually summarize these preclinical findings, Figure 2 illustrates the specific subcellular and molecular effects of creatine supplementation on nerve cells.

**Figure 3:**
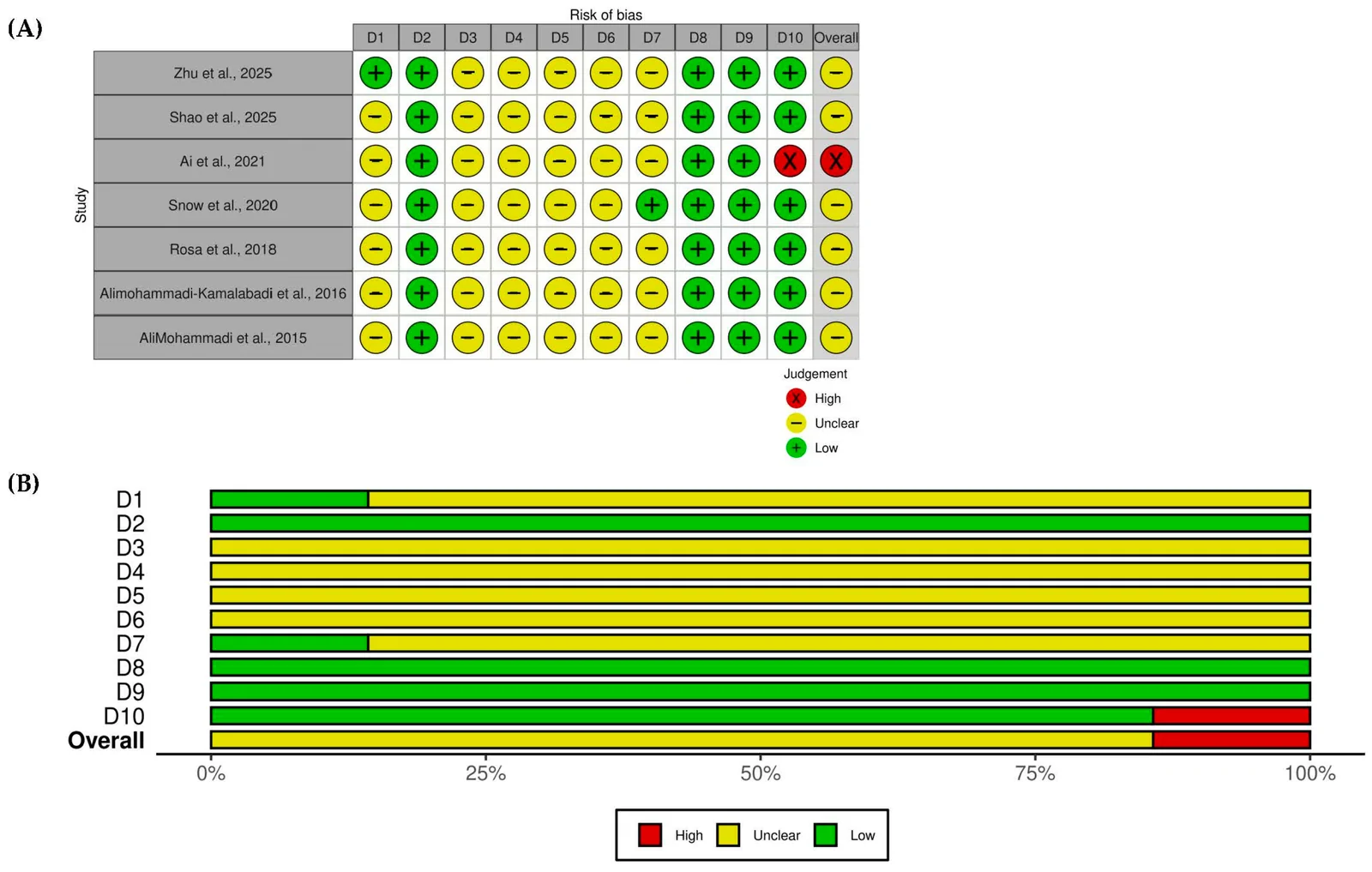
Risk of bias assessment of the included preclinical animal studies utilizing the SYRCLE tool. (**A**) Traffic light plot indicating the domain-level judgments for each individual study [97,98,99,100,101,102,103]; (**B**) Summary bar plot showing the overall proportion of studies with low, unclear, or high risk of bias across each methodological domain. D1: Sequence generation; D2: Baseline characteristics; D3: Allocation concealment; D4: Random housing; D5: Blinding of personnel; D6: Random outcome assessment; D7: Blinding of outcome assessment; D8: Incomplete outcome data; D9: Selective outcome reporting; D10: Other sources of bias. Green (+): Low risk; Yellow (−): Unclear risk; Red (×): High risk; Blue: No information. The ‘Overall’ risk-of-bias rating for each study was derived qualitatively, based on an assessment of the individual methodological domains. Images generated using the robvis web-based tool [96].

Evaluations of methodological quality revealed that the overall risk of bias among the preclinical investigations was predominantly moderate. Apart from one conference abstract exempt from formal appraisal due to inherent format limitations [104], most of the included studies (75%, *n* = 6) were classified as having a moderate or unclear risk of bias [97,99,100,101,102,103]. Only a single study was categorized as presenting a high risk of bias [98]. These ratings were primarily driven by an inherent lack of comprehensive reporting regarding randomization, allocation concealment, and blinding procedures across the experimental literature (see Figure 3).

### 3.2. Clinical Evidence in Patients with Alzheimer’s Disease

A single clinical trial (the CABA cohort, NCT05383833), represented across five published reports, evaluating creatine supplementation in elderly patients diagnosed with AD met the inclusion criteria for this review. Notably, all these reports were produced by the same research group in the United States and stem from the same single-arm pilot trial design. The therapeutic intervention was consistently administered orally via supplemented beverages, utilizing CrM as the exclusive active compound. Furthermore, the dosing regimen and timeframe were rigidly standardized, with patients receiving 20 g/day over an 8-week intervention period. All five reports were published within a very narrow timeframe, encompassing one publication in 2024 [106], three reports in 2025 [107,108,109] and one recent report in 2026 [110].

Collectively, these reports assessed a diverse spectrum of endpoints. Specifically, two reports examined the feasibility of CrM supplementation and cognitive performance concurrently with brain and serum creatine levels [107,108]. Two reports focused on muscle-related outcomes, encompassing handgrip strength, lower extremity strength, and muscle morphology [106,109]. The remaining investigation evaluated systemic bioenergetics, mitochondrial respiration, and markers of oxidative stress [110]. In terms of intervention timing, this clinical trial assessed creatine solely as a therapeutic intervention for symptomatic individuals. Detailed trial characteristics, methodological frameworks, and primary outcomes are summarized in Table 2.

To evaluate the central nervous system (CNS) effects of CrM, Smith et al. [107,108] assessed both brain bioavailability and cognitive function following the 8-week supplementation protocol in patients with AD. The intervention successfully elevated systemic and central creatine concentrations, evidenced by significant increases in both fasting serum and brain creatine levels. Concurrently with this biochemical accumulation, the patients demonstrated cognitive improvements. Quantitative assessments using the National Institutes of Health Toolbox and the Mini-Mental State Examination revealed enhanced performance on global and fluid cognitive composites, alongside improvements in list sorting, oral reading, and flanker tests. Furthermore, these findings successfully established the overall feasibility of the CrM regimen in this clinical population.

Regarding systemic bioenergetics, Taylor et al. [110] assessed mitochondrial function and metabolic markers following the CrM supplementation. High-resolution respirometry revealed sex-specific responses in mitochondrial oxygen consumption rates (OCR). Specifically, female participants exhibited a global enhancement in mitochondrial respiration capacity, evidenced by significantly increased OCR in both platelets and lymphocytes. In contrast, male patients demonstrated no significant alterations in these parameters. CrM supplementation elevated absolute lymphocyte ADP and ATP levels, indicative of increased systemic energy availability, while the overall ATP/ADP ratio remained unchanged. Finally, central markers of neuronal integrity and oxidative defense, specifically N-acetylaspartate (NAA) and GSH quantified via magnetic resonance spectroscopic imaging, were unaffected by the supplementation protocol.

Lastly, regarding physical and functional outcomes, two reports by Smith et al. [106,109] reported significant enhancements in upper body functional capacity, demonstrating increased handgrip strength following CrM supplementation. Conversely, lower extremity strength, assessed via leg dynamometry, remained unchanged [109]. Additionally, while overall body mass index [106] and specific muscle thickness exhibited no significant alterations, the intervention successfully increased the muscle cross-sectional area in the rectus femoris and vastus medialis [109]. This hypertrophic response, coupled with the observed strength gains, was accompanied by a reduction in lower body subcutaneous fat, suggesting an overall improvement in body composition and physical function.

**Figure 4:**
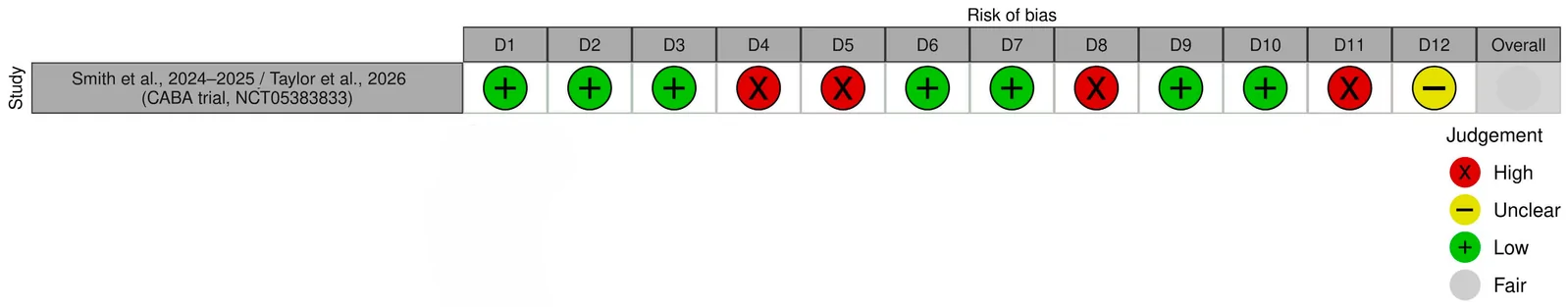
Risk of bias assessment of the included clinical pilot trials utilizing the NIH Quality Assessment Tool for Before-After (Pre-Post) Studies with No Control Group [106,107,108,109,110]. Traffic light plot indicating the item-level judgments for each individual pilot trial. D1: Objective clearly stated; D2: Selection criteria prespecified; D3: Representative participants; D4: All eligible participants enrolled; D5: Sufficient sample size; D6: Intervention clearly described; D7: Outcome measures valid and reliable; D8: Blinding of outcome assessors; D9: Loss to follow-up <20%; D10: Statistical analysis of pre-post changes; D11: Interrupted time-series design; D12: Group-level statistical analysis. Green: low risk of bias; Red: high risk of bias; Yellow: Unclear (cannot determine, not reported, or not applicable); Gray: Fair (moderate risk of bias). The ‘Overall’ risk-of-bias rating for each study was derived qualitatively, based on an assessment of the individual methodological domains. Images generated using the robvis web-based tool [96].

Methodological quality assessments of the clinical trials revealed a fair rating, as illustrated in Figure 4. To avoid duplicate appraisal of the same patient cohort, the risk of bias was assessed by evaluating the CABA trial (NCT05383833) as a unified entity, aggregating methodological details from across all five associated reports [106,107,108,109,110].

## 4. Discussion

### 4.1. Overview of the Current Evidence Landscape

The primary objective of this scoping review was to systematically map the extent, range, and nature of research on creatine supplementation for AD. Despite creatine being a widely recognized and extensively researched compound with a robust theoretical rationale for neuroprotection, our findings reveal a surprising scarcity of empirical evidence within the context of AD. Although preclinical investigations span a decade (2015–2025), the available literature is scarce. Furthermore, the preclinical foundation is weak due to substantial methodological heterogeneity, as the included studies vary considerably in dosing paradigms, administration routes, and targeted outcomes. Consequently, while these animal models generally suggest neuroprotective trends, the overall body of evidence remains fragmented.

Conversely, clinical translation is even more nascent and localized. All the included clinical literature emerged abruptly within the two-year period of 2024–2026, followed the same intervention protocol, and was conducted exclusively in the United States.

Regarding experimental designs, mapped literature reveals a clear preference for therapeutic over preventive interventions. As our results demonstrate, most preclinical models and the human trials administered creatine only after the onset of AD-like pathology or clinical symptoms. This late-stage focus leaves the potential of creatine as an early neuroprotective intervention in asymptomatic or at-risk populations largely unexplored and represents a significant translational gap.

These findings highlight a critical juncture in the field: while the theoretical foundations for creatine’s bioenergetic efficacy are well-established, its experimental consolidation and clinical validation are still in their infancy. Therefore, interpreting the current landscape requires extreme caution, recognizing the gap between neurobiological hypotheses and the current lack of empirical data.

### 4.2. Mechanisms and Translational Efficacy of Creatine Supplementation in Alzheimer’s Disease

**Figure 5:**
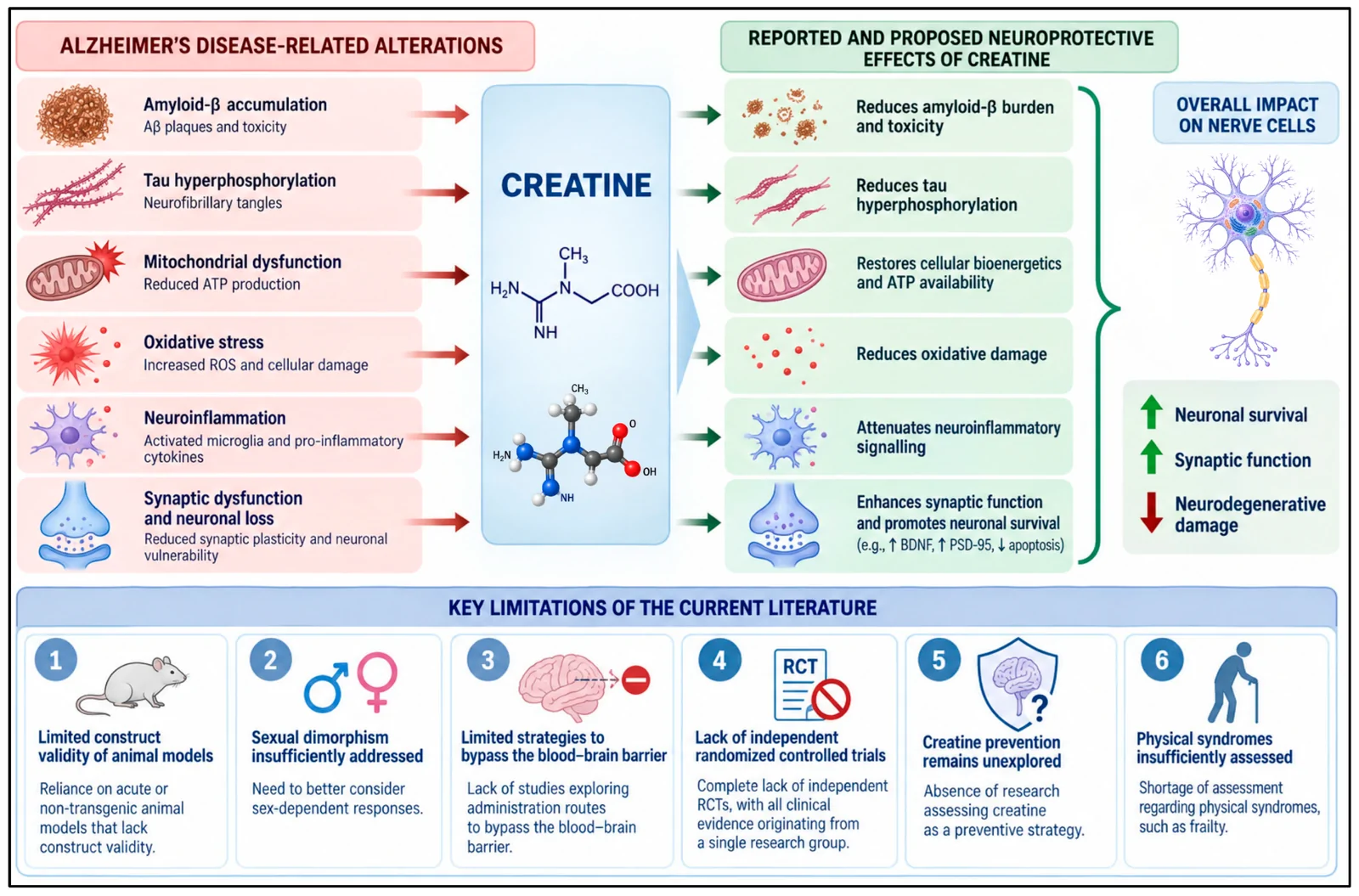
Proposed biological mechanism of creatine supplementation and key limitations of current literature. The arrows indicate: ↓ decreased; ↑ increased. Individual graphical elements were generated using ChatGPT (OpenAI, 2026) and subsequently arranged and composed by the authors using Canva (Canva Pty Ltd., Sydney, Australia).

This article also focuses on synthesizing the current findings and identifying the gaps that still need to be addressed. As outlined in the introduction, the early pathogenesis of AD is fundamentally characterized by a bioenergetic crisis, driven by progressive cerebral glucose hypometabolism and mitochondrial dysfunction. As illustrated in Figure 5, the preclinical evidence reviewed herein suggests that creatine supplementation acts not merely as a symptomatic cognitive enhancer, but as a pleiotropic strategy that targets this underlying metabolic collapse.

At the core of these neuroprotective effects is the restoration of cellular bioenergetics. Experimental and clinical models demonstrated that creatine supplementation significantly enhances mitochondrial coupled respiration [103,110]. These findings are consistent with select in vitro and in vivo studies that demonstrate creatine’s ability to prevent mitochondrial structural damage and promote mitochondrial biogenesis [82,111,112]. Potentially linked is the observation of anti-apoptotic effects, which include the downregulation of pro-apoptotic markers (Caspase-3, Caspase-9, and Bax) and the activation of survival pathways, such as AKT and an increase in p-GSK-3β/GSK-3β ratio [98,99]. This connection could be explained by how preserving mitochondrial integrity directly prevents Bax-mediated membrane permeabilization, which halts the downstream cascade of Caspase-9 and Caspase-3. Additionally, sustained cellular ATP levels may activate energy-dependent survival kinases, such as AKT, which subsequently phosphorylate and deactivate pro-apoptotic targets such as GSK-3β.

Furthermore, this bioenergetic rescue could translate into a potent mitigation of both oxidative stress and neuroinflammation, two hallmark drivers of AD progression. Creatine supplementation consistently augmented endogenous antioxidant defenses while decreasing lipid peroxidation and ROS demand [97,98,99], a property that has been documented since 2002 [113]. Concurrently, it suppressed neuroinflammatory signaling, notably the IFN-γ/STAT1 pathway and microglial activation [100]. In this regard, a comprehensive review by Cordingley et al. [29] highlighted the promising anti-inflammatory potential of creatine supplementation across various pathological conditions and exercise paradigms.

Ultimately, this multi-targeted molecular defense culminated in a reduction in classical AD pathology (Aβ burden and p-Tau expression) and the preservation of synaptic integrity, as reflected by increased structural plasticity markers such as BDNF and PSD-95 [97,98,100,103].

Translating these neurobiological alterations into functional outcomes, various preclinical studies and a human clinical pilot trial have reported notable improvements in cognitive performance and behavioral phenotypes following creatine supplementation [97,99,100,103,107].

### 4.3. Limitations of the Current Evidence

To determine which of these proposed mechanisms warrant further investigation, it is first necessary to acknowledge the overall scarcity of the current literature; inherently, this limitation dictates that all molecular pathways discussed herein remain subject to broader validation. Nevertheless, mechanisms such as the attenuation of oxidative stress and anti-apoptotic protection have been observed across some experimental models. In contrast, data regarding the clearance of classical AD pathology biomarkers yields more heterogeneous results, with some studies failing to replicate these improvements. Finally, while mechanisms involving neuroinflammatory suppression and mitochondrial enhancement show promising therapeutic value, the supporting data remain preliminary and require further experimental replication.

To properly contextualize these varying levels of evidence, a critical appraisal of the findings using the SYRCLE tool reveals notable methodological vulnerabilities, which must be interpreted with nuance. As is widespread in experimental animal literature, a significant proportion of the included studies yielded an “unclear” risk of bias across multiple domains, most notably in allocation concealment, random housing, and blinded outcome assessment. However, it is crucial to acknowledge that these recurrent omissions frequently reflect the historically lenient reporting standards in basic science rather than inherently flawed or biased experimental designs. Within this context of data reporting, it is important to highlight potential inaccuracies within the primary literature. For instance, the preclinical study by Ai et al. [98] explicitly reports using “Sprague-Dawley mice” weighing “20 kg”. While the weight is a physiological impossibility and likely a typographical error (presumably intended as 20 g), the strain confusion leaves the exact animal species uncertain.

Beyond methodological reporting, the construct validity of the experimental models utilized warrants consideration. A substantial proportion of the included preclinical studies relied on non-transgenic approximations of AD, specifically D-galactose-induced aging models and acute Aβ injections. While these paradigms are useful for investigating isolated neurotoxic or oxidative mechanisms, they do not fully recapitulate the chronic, progressive, and multifactorial pathogenesis of human AD. As a result, extrapolating these specific neuroprotective findings to the human clinical condition requires caution. Although studies employing transgenic models (such as the 3xTg and FAD4T lines) provide a more accurate representation of the disease, future preclinical research would benefit from prioritizing these authentic models to enhance translational reliability.

Also important to the construct validity of the experimental model is the consideration of intrinsic biological variables. Most notably, some studies have reported a lack of therapeutic efficacy in males [101,102]. However, because these specific studies lacked a female comparison group, it remains unclear whether this represents a true sex-dependent effect. To date, only Snow et al. [103] has directly assessed sex-specific responses in this context, observing cognitive improvements in females alongside altered spatial search strategies in males. Remarkably, this sexual dimorphism has been observed previously; preclinical studies in depression have shown a lack of behavioral response, and even paradoxical symptom exacerbation, in male rodents [114,115]. This divergence is mirrored in human epidemiological data, where the protective association of dietary creatine against depression is highly significant in females but completely absent in males [116].

Alongside these sex-dependent responses, another critical methodological factor that could determine creatine’s therapeutic efficacy is the dose and route of administration. Although chronic supplementation via a 3% supplemented diet successfully elicited molecular and behavioral benefits, protocols utilizing a lower 2% dose failed to reverse Aβ plaque accumulation, prevent neuronal apoptosis, or rescue cognitive deficits [101,102]. This discrepancy is significant because it suggests that a high concentration of systemic creatine is necessary to cross the BBB and achieve therapeutic saturation in the CNS. However, inferences regarding dose–response and BBB saturation are relative given the existing differences in species, models, and protocols across the available literature. For instance, Snow et al. [103] reported cognitive improvements in females without significant changes in hippocampal creatine levels, suggesting alternative systemic compensatory mechanisms rather than direct CNS saturation. Despite these uncertainties, the route of administration remains a relevant variable to consider. While most of the studies included in this review used oral supplementation to accurately mirror standard human clinical protocols, this approach is inherently limited by transporter saturability. Only one preclinical study included in this review employed a route capable of bypassing the BBB entirely, highlighting this anatomical hurdle. Using a single direct intracerebroventricular injection, this intervention produced pronounced neuroprotective and cognitive outcomes [100]. Given these results, the absence of studies exploring intranasal administration represents a notable gap. Intranasal delivery offers a compelling, non-invasive strategy to bypass the BBB directly via the olfactory and trigeminal neural pathways. Recent evidence has already validated the feasibility and enhanced brain penetrance of intranasal creatine supplementation in rats [117], highlighting it as a highly translational avenue for future research. Additionally, while creatine possesses a widely documented renal safety profile in healthy populations [2,5,6,10], the clinical safety of high-dose protocols aimed at maximizing brain exposure should be carefully evaluated in older AD patients.

In parallel, a critical appraisal of clinical literature reveals a substantial evidence bias stemming from a complete lack of independent replication. The clinical evidence stems entirely from a single research group reporting on one non-controlled, single-arm pilot trial cohort over an eight-week period. Using the NIH Quality Assessment Tool for Before-After Studies with No Control Group, this investigation was rated as “fair” in terms of methodology. While this trial reports encouraging outcomes, including improvements in global and fluid cognitive composites, increased upper-body strength, and sex-specific enhancements in mitochondrial respiration, its findings must be interpreted with caution [106,107,108,109,110]. The primary limitations, small sample sizes and lack of a randomized placebo control group, make it difficult to definitively isolate the therapeutic effects of creatine from confounding variables. Thus, while this clinical landscape shows the feasibility and tolerability of the intervention, it also highlights that its definitive therapeutic efficacy in patients with AD must be established through robust, randomized, controlled trials. Furthermore, the clinical scope of this trial remains severely constrained. Current protocols have exclusively evaluated creatine as a late-stage therapeutic intervention in symptomatic patients, leaving its potential as an early, preventive strategy in asymptomatic or at-risk populations entirely unexplored. Similarly, while muscle strength has been assessed, there is a notable absence of research investigating broader, highly relevant geriatric physical syndromes, such as sarcopenia and frailty, which frequently co-occur with cognitive decline.

In summary, based on the mapped literature, we have identified six limitations within the current evidence that restrict translational progress: (1) a reliance on acute or non-transgenic animal models; (2) a need to address sexual dimorphism; (3) a lack of studies exploring administration routes to bypass the blood–brain barrier; (4) a complete lack of independent RCTs, with all clinical evidence originating from a single research group; (5) an absence of research assessing creatine as a preventive strategy; and (6) a shortage of assessment regarding physical syndromes, such as frailty.

### 4.4. Methodological Limitations of the Review

Some limitations of the methodology of this scoping review must be acknowledged. First, our search strategy was designed to maximize specificity for AD, excluding broader terms such as “dementia”, related compounds like “phosphocreatine”, and studies assessing AD as a secondary outcome. While this approach ensured pathological homogeneity among the included studies, it could reduce the overall sensitivity of the search. Second, although our eligibility criteria included four specific languages (English, Spanish, Portuguese, or Italian), the search strings were constructed exclusively in English. Consequently, most retrieved and ultimately included literature was published in English, which introduces a potential language bias. Finally, in strict adherence to PRISMA-ScR transparency norms, we must acknowledge that the OSF protocol for this review was registered retrospectively in March 2026, after the first literature search was conducted in October 2025.

### 4.5. Future Directions

Considering the current limitations, several avenues for future research are proposed to further elucidate the translational potential of creatine in AD.

First, to improve the construct validity of preclinical findings, future investigations should prioritize the use of progressive, transgenic models of AD over acute neurotoxicity or generalized aging paradigms.

Second, exploring alternative, non-invasive routes of administration could be interesting. For example, given the saturability of BBB transporters with oral supplementation, investigating the intranasal delivery of creatine offers a practical approach to achieve direct CNS penetrance via the olfactory and trigeminal pathways.

Third, the observation of sexual dimorphism highlights the need to account for sex as a biological variable in experimental designs. Future investigations in both animal models and human trials should aim to include both sexes and report disaggregated data. This approach is necessary to better understand the divergent neurobiological responses between males and females and to adapt therapeutic protocols accordingly.

Fourth, from a clinical perspective, it would be useful to broaden the scope of physical assessments. While the preliminary pilot trial has reported localized improvements in muscle strength and size, future studies could evaluate comprehensive geriatric syndromes, specifically sarcopenia and physical frailty. Given the known effects of creatine on age-related physical decline in healthy older adults [118,119], investigating its impact on frailty indices in AD patients may offer a dual benefit by addressing both cognitive and functional decline.

Fifth, future research should transition from evaluating creatine only as a therapeutic treatment to exploring its potential as a preventive strategy. Given that AD pathobiology develops across a silent biological continuum decades before clinical symptoms manifest, early bioenergetic interventions may yield significantly higher efficacy when deployed in asymptomatic or at-risk individuals. Indeed, the current literature underscores that preventive neuroprotective interventions in at-risk patients represent one of the most promising avenues for creatine research [24,46]. Preclinical and clinical designs should therefore investigate long-term creatine supplementation regimens initiated during middle age or at the earliest stages of mild cognitive impairment to rigorously evaluate its capacity to delay or prevent the onset of AD neuropathology.

Finally, the clinical evaluation of creatine should advance from uncontrolled pilot studies to adequately powered, double-blind, randomized placebo-controlled trials to more accurately determine its therapeutic efficacy in AD.

## 5. Conclusions

This scoping review reveals that, although there is a strong theoretical rationale for the neuroprotective role of creatine in AD, the current empirical evidence is still in its early stages and fragmented. Preclinical studies suggest that creatine supplementation exerts antioxidant and anti-apoptotic effects and contributes to the restoration of cellular bioenergetics. However, data on the reduction of classical pathological biomarkers and the reversal of cognitive deficits show significant heterogeneity, influenced by factors such as dosage and sexual dimorphism. Additionally, mitigating neuroinflammation and enhancing mitochondrial function emerge as other promising mechanistic targets.

Concurrently, clinical evidence in humans is limited to a single uncontrolled, single-arm pilot trial. While this initial trial suggests the feasibility and tolerability of the intervention, methodological limitations prevent definitive conclusions about therapeutic efficacy.

In conclusion, substantial further research is needed to clarify the translational potential of creatine in this context. Future investigations must prioritize authentic AD models, consider sex as a biological variable, evaluate comprehensive geriatric syndromes such as sarcopenia and physical frailty, explore alternative non-invasive administration routes such as intranasal delivery to address BBB limitations, assess creatine supplementation as a preventive strategy, and progress toward randomized, double-blind, placebo-controlled clinical trials.

## Figures and Tables

**Table 1:** Study characteristics and key findings of creatine supplementation in preclinical animal models of Alzheimer’s disease.

Author and Year [Ref]	Animal Species and Model (Sex, Age)	Intervention Timing, Duration and Groups (*n*)	Administration Route (Dose)	Outcomes Measured (Tool)	Main Results (Implications)	Risk of Bias
Zhu et al., 2025 [97]	C57BL/6J mice D-Gal-Induced Aging Model (♂, -)	Preventive (8 weeks) (1) Control (15) (2) D-gal (15) (3) Cr (15) (4) Cr + D-gal (15)	Oral via diet (3% supplemented)	Short-term memory (Y-maze) Spatial learning and memory capabilities (MWM) SOD, GSH and MDA (Assay) PSD-95, NF-L and BDNF (Western blot) CK-BB activity (Assay) Dendritic branching patterns and spines (Golgi–Cox Staining)	Cr supplementation: ↔ swim speed, ↓ escape latency, ↑ time in target quadrant, ↑ number of passes over platform, ↑ entry ratio (↓ cognitive deficits) ↑ SOD and GSH-Px activity and ↓ MDA level (↓ oxidative stress) ↑ PSD-95, NF-L and BDNF (↑ structural plasticity of hippocampal neurons) ↑ activity and protein levels of CK-BB ↑ spine density and total dendritic length of hippocampal neurons in CA1	Moderate/Unclear risk
Shao et al., 2025 [100]	FAD4T [B6/JGpt-Tg (ThyAPP/Thy-PSEN1)-5/Gpt Transgenic Mouse (♀, 10–11 months)	Therapeutic (4 weeks) (1) Cr (6) (2) Phosphate-Buffered Saline (5)	Intraventricular Injection, once weekly (0.125 mg)	Spatial learning and memory capabilities (MWM) STAT1, pSTAT1, β-actin (Western Blot) pSTAT1, NeuN, Aβ, IBA1, GFAP (Immunofluorescence Staining)	Cr supplementation: ↓ escape latency, ↑ time in target quadrant (↑ spatial memory) ↓ Aβ (↓ AD-associated molecular pathology) ↓ IBA1, ↓ pSTAT1 (↓ neuroinflammation) ↑ GFAP (↑ astrocytic support) ↓ IFN-γ/STAT1 signaling pathway (↑ Neuroprotection)	Moderate/Unclear risk
Ai et al., 2021 [98]	Mice D-Gal-Induced Aging Model (♂, -) *	Preventive (10 weeks) (1) Control (8) (2) D-gal (8) (3) PCr 50 mg/kg (8) (4) PCr 20 mg/kg + D-gal (8) (5) PCr 50 mg/kg + D-gal (8)	PCr injection once a week for last 8 weeks (50 mg/kg or 20 mg/kg)	Apoptosis and Morphology (TUNEL staining, H&E staining) Bcl-2/Bax, Caspase-3, Caspase-9, p-AKT/AKT, p-GSK-3β/GSK-3β, p-Tau/Tau, APP, CDK5 (Western Blot) SOD, NO, MDA (Biochemical Assay Kits)	PCr supplementation: ↓ neuronal damage (pyknosis/shrinkage) in CA1/CA3. (↓ apoptosis) ↓ ratio of green fluorescence (↓ apoptosis) ↑ Bcl-2/Bax ↓ Caspase-3, Caspase-9 (↓ apoptosis) ↑ p-AKT/AKT, p-GSK-3β/GSK-3β (↑ survival pathway) ↓ p-Tau/Tau, APP, CDK5 (↓ AD-associated molecular pathology) ↑ SOD, NO ↓ MDA (↓ oxidative stress)	High risk
Snow et al., 2020 [103] (includes Snow et al., 2014 [105])	3xTg mice AD model (♂/♀, 7 months)	Therapeutic (8 weeks) (1) Control ♂ (6) (2) Control ♀ (6) (3) Cr ♂ (5) (4) Cr ♀ (8)	Oral via diet (3% supplemented)	Spatial learning and memory capabilities (MWM) Hippocampal Cr levels (Colorimetric Assay) Hippocampal Mitochondrial Oxygen Consumption (Seahorse Analyzer) CREB, pCREB, IκBα, CaMKII, PSD-95, Aβ, APP, tau, ptau, Drp1, Porin, Complex 1. (Western Blot)	Cr supplementation: ♀ ↑ swim speed, ↓ escape latency, ↑ time in target quadrant, ↑ number of passes over platform, ↑ entry ratio (↓ cognitive deficits) ♂ ↓ spatial search strategies ♀ ↔ hippocampal Cr levels ↑ coupled respiration and coupling efficiency (↑ mitochondrial function and ATP production) ♀ ↑ pCREB/CREB, IkBα, CaMKII, PSD-95 (↑ synaptic plasticity and ↓ neuroinflammation) ↓ A β Trimers (↓ neurotoxicity and associated cognitive deficits)	Moderate/Unclear risk
Rosa et al., 2018 [99]	Aβ1–40 Intraventricularly Injected Swiss mice (♀, 1.5–2 months)	Therapeutic (Single dose) (1) Vehicle + Vehicle (8) (2) Vehicle + Cr 0.01 mg/kg (8) (3) Vehicle + Cr 10 mg/kg (8) (4) Vehicle + Fluoxetine (8) (5) Aβ + Vehicle (8) (6) Aβ + Cr 0.01 mg/kg (8) (7) Aβ + Cr 10 mg/kg (8) (8) Aβ + Fluoxetine (8)	Oral via gavage (0.01 mg/kg or 10 mg/kg)	Depressive behavior (Tail suspension test, Splash test, Open field test) pGSK-3β, GSK-3β, HO-1 (Western Blot) GSH levels (fluorometric assay) GR and TrxR (spectrophotometric assays)	Cr supplementation: 0.01 mg/kg and 10 mg/kg: ↓ immobility time, ↑ grooming time and ↓ latency to groom (↓ depressive behavior and anhedonia) 0.01 mg/kg: ↑ pGSK-3β and HO-1 (↑ antioxidant defenses) 0.01 mg/kg and 10 mg/kg: ↓ GSH, GR, TrxR (↓ reactive antioxidant demand)	Moderate/Unclear risk
Alimohammadi-Kamalabadi et al., 2016 [101]	Bilateral intrahippocampal Aβ1–42 peptide injected Wistar rats (♂, 4 months)	Preventive (10 weeks, pre and post-surgery) (1) Control (8) (2) Sham (8) (3) Aβ (8) (4) Aβ + Cr (8)	Oral via diet (2% supplemented)	Spatial learning and memory capabilities (MWM) Apoptotic neurons and Aβ plaques in CA1 (TUNEL assay and Thioflavin-T staining, respectively)	Cr supplementation: ↑ escape latency, ↓ time in target quadrant, ↑ mean distance traveled (↑ cognitive deficits) ↔ apoptotic neurons ↔ Aβ plaques	Moderate/Unclear risk
AliMohammadi et al., 2015 [102]	Bilateral intrahippocampal Aβ1–42 peptide injected Wistar rats (♂, -)	Therapeutic (6 weeks) (1) Control (8) (2) Sham (8) (3) Aβ (8) (4) Aβ + Cr (8)	Oral via diet (2% supplemented)	Spatial learning and memory capabilities (MWM) Apoptotic neurons and Aβ plaques in CA1 (TUNEL assay and Thioflavin-T staining, respectively)	Cr supplementation: ↔ escape latency, ↔ time in target quadrant, ↔ mean distance traveled (↔ cognitive deficits) ↔ apoptotic neurons ↔ Aβ plaques	Moderate/Unclear risk
Farshidfar et al., 2015 [104]	3xTg mice AD model (♂/♀, 8 months)	Therapeutic (8 weeks) (1) ♂ Control (6) (2) ♀ Control (6) (3) ♂ Cr (6) (4) ♀ Cr (6)	Oral via diet 3% weight for weight (*w*/*w*)	Body weight and feed intake Creatine content and amino acid levels in quadriceps muscle (High-performance liquid chromatography)	Cr supplementation: ↔ body weight and feed intake ↑ creatine content, ↑ total BCAA levels, ↑ BCAA/non-BCAA ratio, ↑ leucine levels (↑ muscle function)	N/A

Study characteristics and primary outcomes of creatine supplementation in preclinical animal models of Alzheimer’s disease. This table summarizes the study designs, behavioral outcomes, and molecular mechanisms associated with creatine or phosphocreatine administration. Abbreviations: (Aβ): amyloid beta; (AD): Alzheimer’s disease; (AKT): protein kinase B; (APP): amyloid precursor protein; (Bax): Bcl-2-associated X protein; (BCAA): branched-chain amino acids; (Bcl-2): B-cell lymphoma 2; (BDNF): brain-derived neurotrophic factor; (CaMKII): calmodulin-dependent protein kinase II; (CDK5): cyclin-dependent kinase 5; (CK-BB): creatine kinase brain type; (Cr): creatine; (CREB): cAMP response element-binding protein; (D-gal): D-galactose; (Drp1): dynamin-related protein 1; (GFAP): glial fibrillary acidic protein; (GR): glutathione reductase; (GSH): glutathione; (GSH-Px): glutathione peroxidase; (GSK-3β): glycogen synthase kinase 3 beta; (H&E): hematoxylin and eosin; (HO-1): heme oxygenase-1; (HPLC): high-performance liquid chromatography; (IBA1): ionized calcium-binding adapter molecule 1; (IFN-γ): interferon gamma; (IκBα): nuclear factor of kappa light polypeptide gene enhancer in B-cells inhibitor, alpha; (MDA): malondialdehyde; (MWM): Morris water maze; (NeuN): neuronal nuclei; (NF-L): neurofilament light chain; (NO): nitric oxide; (NR): not reported; (PCr): phosphocreatine; (pCREB): phosphorylated CREB; (p-GSK-3β): phosphorylated GSK-3β; (PSD-95): postsynaptic density protein 95; (pSTAT1): phosphorylated STAT1; (p-Tau): phosphorylated tau; (SOD): superoxide dismutase; (STAT1): signal transducer and activator of transcription 1; (TrxR): thioredoxin reductase; (TUNEL): terminal deoxynucleotidyl transferase dUTP nick end labeling. The arrows indicate: ↓ decreased; ↔ no significant change or effect; ↑ increased. N/A: Not applicable due to conference abstract format limiting formal bias appraisal. The symbols ♀ and ♂ indicate female and male, respectively. In main results, text in parentheses denotes the functional implication or biological interpretation of the observed outcomes. * The original authors explicitly reported using “Sprague-Dawley (SD) mice” weighing “20 ± 2 kg”. We report this verbatim to preserve extraction fidelity, although Sprague-Dawley is standardly a rat strain and the reported weight is biologically anomalous for rodents.

**Table 2:** Study characteristics and primary outcomes of creatine supplementation in patients with Alzheimer’s disease.

Author and Year [Ref]	Country	Study Design (Population)	Intervention Timing, Duration and Groups (*n*)	Administration Route (Dose)	Outcomes Measured (Tool)	Main Results (Implications)	Risk of Bias
Smith et al., 2024–2025/Taylor et al., 2026 [106,107,108,109,110] (CABA trial, NCT05383833)	USA	Single-arm pilot trial (73.1 ± 6.3 years old AD patients)	Therapeutic (8 weeks) (1) ♀ CrM (7) (2) ♂ CrM (13)	Oral beverages (20 g/day)	Fasting serum Cr (Clinical laboratory analysis) Brain Cr as a ratio to unsuppressed water (magnetic resonance spectroscopy) Cognition (National Institutes of Health Toolbox, Mini-Mental State Examination) Body mass index Handgrip strength Lower body strength (leg dynamometry) Muscle size cross-sectional area in rectus femoris and vastus medialis (mCSA) Muscle thickness in rectus femoris, vastus medialis, and vastus lateralis. Plasma C-terminal agrin fragment levels (NMJ integrity) GSH and NAA (1H magnetic resonance spectroscopic imaging) Mitochondrial OCR in platelets and lymphocytes (Oroboros Oxygraph-2k) ADP and ATP levels in lymphocytes (Bioluminescent assay) Lymphocyte ROS (Flow cytometry)	Cr supplementation: feasible ↑ serum Cr levels ↑ brain Cr levels ↑ cognition on global and fluid composites, list sorting, oral reading and flanker tests (↓ cognitive deficits) ↔ body mass index ↑ handgrip strength (↑ muscle strength) ↑ handgrip strength (↑ muscle strength) ↔ lower body strength (↔ muscle strength) ↑ muscle size ↔ muscle thickness ↓ subcutaneous fat in lower body (↑ body function) ↔ C-terminal agrin fragment ♂ ↔ platelet and lymphocyte OCR. ♀ ↑ platelet OCR and ↑ lymphocyte OCR (↑ global enhancement of mitochondrial respiration capacity). ↔ NAA and GSH ↑ lymphocyte ADP and ATP, ↔ ATP/ADP ratio (↑ systemic energy availability)	Fair

Characteristics and main findings of the included human clinical studies on creatine intervention. This table summarizes the study designs, physical and cognitive outcomes, and bioenergetic mechanisms associated with creatine monohydrate supplementation in human participants. Abbreviations: (AD): Alzheimer’s disease; (ADP): adenosine diphosphate; (ATP): adenosine triphosphate; (Cr): creatine; (CrM): creatine monohydrate; (GSH): glutathione; (mCSA): muscle cross-sectional area; (NAA): N-acetylaspartate; (NMJ): neuromuscular junction; (OCR): oxygen consumption rate; (ROS): Reactive Oxygen Species; (USA): United States of America. The arrows indicate: ↓ decreased; ↔ no significant change or effect; ↑ increased. The symbols ♀ and ♂ indicate female and male, respectively. In main results, text in parentheses denotes the functional implication or biological interpretation of the observed outcomes.

## Data Availability

No new data were created or analyzed in this study. Data sharing is not applicable to this article.

## Supplementary Materials

The following supporting information can be downloaded at https://www.mdpi.com/article/10.3390/nu18213477/s1, Document Supplementary Materials: S1. PRISMA-ScR Checklist; S2. Full Search Strategies; S3. Detailed Risk of Bias Assessment Tables.

## Abbreviations

The following abbreviations are used in this manuscript:

Aβ	Amyloid-beta
AD	Alzheimer’s disease
ADP	Adenosine diphosphate
ATP	Adenosine triphosphate
Bax	Bcl-2-associated X protein
BBB	Blood–brain barrier
BDNF	Brain-derived neurotrophic factor
CNS	Central nervous system
CrM	Creatine monohydrate
D-gal	D-galactose
GFAP	Glial fibrillary acidic protein
GSH	Glutathione
GSK-3β	Glycogen synthase kinase 3 beta
IBA1	Ionized calcium-binding adapter molecule 1
JBI	Joanna Briggs Institute
mCSA	Muscle cross-sectional area
MDA	Malondialdehyde
MWM	Morris water maze
NAA	N-acetylaspartate
NFTs	Neurofibrillary tangles
NIH	National Institutes of Health
NMJ	Neuromuscular junction
OCR	Oxygen consumption rate
PCC	Population, Concept, and Context
PCr	Phosphocreatine
PRISMA	Preferred Reporting Items for Systematic Reviews and Meta-Analyses
PSD-95	Postsynaptic density protein 95
RCTs	Randomized controlled trials
ROS	Reactive oxygen species
SOD	Superoxide dismutase
STAT1	Signal transducer and activator of transcription 1
SYRCLE	Systematic Review Centre for Laboratory animal Experimentation
