# Supplementary material for: A Scoping Review of Creatine Supplementation in Alzheimer’s Disease: Evidence from Preclinical and Clinical Studies
Source: Nutrients. 2026 Oct 23;18(21):3477. doi: 10.3390/nu18213477 (PMC13764930; doi:10.3390/nu18213477)
Supplement: Supplementary file 1 [file nutrients-18-03477-s001.zip › nutrients-4541015-supplementary.pdf]

SUPPLEMENTARY MATERIALS

S1. PRISMA Checklist Extension for Scoping Review (PRISMA-ScR)

Preferred Reporting Items for Systematic reviews and Meta-Analyses extension for Scoping Reviews (PRISMA-ScR) Checklist

SECTION	ITEM	PRISMA-ScR CHECKLIST ITEM	REPORTED ON PAGE #
**TITLE**			
Title	1	Identify the report as a scoping review.	1
**ABSTRACT**			
Structured summary	2	Provide a structured summary that includes (as applicable): background, objectives, eligibility criteria, sources of evidence, charting methods, results, and conclusions that relate to the review questions and objectives.	1
**INTRODUCTION**			
Rationale	3	Describe the rationale for the review in the context of what is already known. Explain why the review questions/objectives lend themselves to a scoping review approach.	2
Objectives	4	Provide an explicit statement of the questions and objectives being addressed with reference to their key elements (e.g., population or participants, concepts, and context) or other relevant key elements used to conceptualize the review questions and/or objectives.	4
**METHODS**			
Protocol and registration	5	Indicate whether a review protocol exists; state if and where it can be accessed (e.g., a Web address); and if available, provide registration information, including the registration number.	4
Eligibility criteria	6	Specify characteristics of the sources of evidence used as eligibility criteria (e.g., years considered, language, and publication status), and provide a rationale.	4
Information sources*	7	Describe all information sources in the search (e.g., databases with dates of coverage and contact with authors to identify additional sources), as well as the date the most recent search was executed.	5
Search	8	Present the full electronic search strategy for at least 1 database, including any limits used, such that it could be repeated.	5
Selection of sources of evidence†	9	State the process for selecting sources of evidence (i.e., screening and eligibility) included in the scoping review.	5
Data charting process‡	10	Describe the methods of charting data from the included sources of evidence (e.g., calibrated forms or forms that have been tested by the team before their use, and whether data charting was done independently or in duplicate) and any processes for obtaining and confirming data from investigators.	5
Data items	11	List and define all variables for which data were sought and any assumptions and simplifications made.	5
Critical appraisal of individual sources of evidence§	12	If done, provide a rationale for conducting a critical appraisal of included sources of evidence; describe the methods used and how this information was used in any data synthesis (if appropriate).	5
Synthesis of results	13	Describe the methods of handling and summarizing the data that were charted.	6
**RESULTS**			
Selection of sources of evidence	14	Give numbers of sources of evidence screened, assessed for eligibility, and included in the review, with reasons for exclusions at each stage, ideally using a flow diagram.	6
Characteristics of sources of evidence	15	For each source of evidence, present characteristics for which data were charted and provide the citations.	7
Critical appraisal within sources of evidence	16	If done, present data on critical appraisal of included sources of evidence.	12,15
Results of individual sources of evidence	17	For each included source of evidence, present the relevant data that were charted that relate to the review questions and objectives.	7
Synthesis of results	18	Summarize and/or present the charting results as they relate to the review questions and objectives.	7
**DISCUSSION**			
Summary of evidence	19	Summarize the main results (including an overview of concepts, themes, and types of evidence available), link to the review questions and objectives, and consider the relevance to key groups.	15
Limitations	20	Discuss the limitations of the scoping review process.	16
Conclusions	21	Provide a general interpretation of the results with respect to the review questions and objectives, as well as potential implications and/or next steps.	19
**FUNDING**			
Funding	22	Describe sources of funding for the included sources of evidence, as well as sources of funding for the scoping review. Describe the role of the funders of the scoping review.	20

JBI = Joanna Briggs Institute; PRISMA-ScR = Preferred Reporting Items for Systematic reviews and Meta-Analyses extension for Scoping Reviews.
* Where *sources of evidence* (see second footnote) are compiled from, such as bibliographic databases, social media platforms, and Web sites.
† A more inclusive/heterogeneous term used to account for the different types of evidence or data sources (e.g., quantitative and/or qualitative research, expert opinion, and policy documents) that may be eligible in a scoping review as opposed to only studies. This is not to be confused with *information sources* (see first footnote).
‡ The frameworks by Arksey and O'Malley (6) and Levac and colleagues (7) and the JBI guidance (4, 5) refer to the process of data extraction in a scoping review as data charting.
§ The process of systematically examining research evidence to assess its validity, results, and relevance before using it to inform a decision. This term is used for items 12 and 19 instead of "risk of bias" (which is more applicable to systematic reviews of interventions) to include and acknowledge the various sources of evidence that may be used in a scoping review (e.g., quantitative and/or qualitative research, expert opinion, and policy document).

From: Tricco AC, Lillie E, Zarin W, O'Brien KK, Colquhoun H, Levac D, et al. PRISMA Extension for Scoping Reviews (PRISMAScR): Checklist and Explanation. Ann Intern Med. 2018;169:467–473. doi: 10.7326/M18-0850.

S2. Full Search Strategies

Full Search Strategy for Embase (via Elsevier)

```
('alzheimer disease'/exp OR 'alzheimer*':ti,ab) AND ('creatine'/exp OR 'creatine':ti,ab OR 'creatine monohydrate':ti,ab OR 'creatine supplementation':ti,ab)
```

Date searched: October 16, 2025

Results: 756

Date searched: October 16, 2025 - August 14, 2026

New results: 53

Full Search Strategy for PubMed (via NCBI)

```
("Alzheimer Disease"[MeSH Terms] OR "Alzheimer*"[tiab] OR "Alzheimer's disease"[tiab]) AND ("Creatine"[MeSH Terms] OR "creatine"[tiab] OR "creatine monohydrate"[tiab] OR "creatine supplementation"[tiab])
```

Date searched: October 16, 2025

Results: 409

Date searched: October 16, 2025 - August 14, 2026

New results: 17

Full Search Strategy for Scopus (via Elsevier)

```
("Alzheimer*" OR "Alzheimer's disease") AND ("creatine" OR "creatine monohydrate" OR "creatine supplementation")
```

Date searched: October 16, 2025

Results: 969

Date searched: October 16, 2025 - August 14, 2026

New results: 82

Full Search Strategy for Web of Science (Core Collection, via Clarivate)

```
("Alzheimer*" OR "Alzheimer's disease") AND ("creatine" OR "creatine monohydrate" OR "creatine supplementation")
```

Date searched: October 16, 2025

Results: 1280

Date searched: October 16, 2025 - August 14, 2026

New results: 60

Full Search Strategy for PsycINFO (via ProQuest)

```
("Alzheimer*" OR "Alzheimer's disease") AND ("creatine" OR "creatine monohydrate" OR "creatine supplementation")
```

Date searched: October 16, 2025

Results: 171

Date searched: October 16, 2025 - August 14, 2026

New results: 2

The final search strategies for each database were selected after testing several combinations of terms in order to obtain the largest possible number of relevant results while maintaining a focused scope.

S3. Detailed Risk of Bias Assessment Tables

NIH Quality Assessment Tool for Before-After (Pre-Post) Studies with No Control Group for pilot trials in humans

Criteria	Yes	No	Other (CD, NR, NA)*
	Smith et al., 2024–2025 / Taylor et al., 2026 [107-111] (CABA trial, NCT05383833)	Smith et al., 2024–2025 / Taylor et al., 2026 [107-111] (CABA trial, NCT05383833)	Smith et al., 2024–2025 / Taylor et al., 2026 [107-111] (CABA trial, NCT05383833)
1. Was the study question or objective clearly stated?			
2. Were eligibility/selection criteria for the study population prespecified and clearly described?			
3. Were the participants in the study representative of those who would be eligible for the test/service/intervention in the general or clinical population of interest?			
4. Were all eligible participants that met the prespecified entry criteria enrolled?			
5. Was the sample size sufficiently large to provide confidence in the findings?			
6. Was the test/service/intervention clearly described and delivered consistently across the study population?			
7. Were the outcome measures prespecified, clearly defined, valid, reliable, and assessed consistently across all study participants?			
8. Were the people assessing the outcomes blinded to the participants' exposures/interventions?			
9. Was the loss to follow-up after baseline 20% or less? Were those lost to follow-up accounted for in the analysis?			
10. Did the statistical methods examine changes in outcome measures from before to after the intervention? Were statistical tests done that provided p values for the pre-to-post changes?			
11. Were outcome measures of interest taken multiple times before the intervention and multiple times after the intervention (i.e., did they use an interrupted time-series design)?			
12. If the intervention was conducted at a group level (e.g., a whole hospital, a community, etc.) did the statistical analysis take into account the use of individual-level data to determine effects at the group level?			

*CD, cannot determine; NA, not applicable; NR, not reported

SYRCLE's tool for assessing risk of bias

Item	Type of bias	Domain	Yes							No							Unclear						
			Zhu et al., 2025	Shao et al., 2025	Ai et al., 2021	Snow et al., 2020	Rosa et al., 2018	Alimo hammadi et al., 2016	AliMo hammadi et al., 2015	Zhu et al., 2025	Shao et al., 2025	Ai et al., 2021	Snow et al., 2020	Rosa et al., 2018	Alimo hammadi et al., 2016	AliMo hammadi et al., 2015	Zhu et al., 2025	Shao et al., 2025	Ai et al., 2021	Snow et al., 2020	Rosa et al., 2018	Alimo hammadi et al., 2016	AliMo hammadi et al., 2015
1	Selection bias	Sequence generation																					
2	Selection bias	Baseline characteristics																					
3	Selection bias	Allocation concealment																					
4	Performance bias	Random housing																					
5	Performance bias	Blinding																					
6	Detection bias	Random outcome assessment																					
7	Detection bias	Blinding																					
8	Attrition bias	Incomplete outcome data																					
9	Reporting bias	Selective outcome reporting																					
10	Other	Other sources of bias																					
